# Temporal Patterns and Drug Resistance in CSF Viral Escape Among ART-Experienced HIV-1 Infected Adults

**DOI:** 10.1097/QAI.0000000000001362

**Published:** 2017-05-16

**Authors:** Shibani S. Mukerji, Vikas Misra, David Lorenz, Anna M. Cervantes-Arslanian, Jennifer Lyons, Spyridon Chalkias, Alysse Wurcel, Deirdre Burke, Nagagopal Venna, Susan Morgello, Igor J. Koralnik, Dana Gabuzda

**Affiliations:** * Department of Cancer Immunology and Virology, Dana-Farber Cancer Institute, Boston, MA;; † Department of Neurology, Massachusetts General Hospital, Boston, MA;; ‡ Departments of Neurology and Neurosurgery, Boston Medical Center, Boston, MA;; § Department of Neurology, Brigham and Womens Hospital, Boston, MA;; ‖ Division of NeuroImmunology, Beth Israel Deaconess Medical Center, Boston, MA;; ¶ Department of Geographic Medicine and Infectious Diseases, Tufts Medical Center, Boston, MA;; # Department of Neurology, Icahn School of Medicine at Mount Sinai, New York, NY; and; ** Department of Neurological Sciences, Rush University Medical Center, Chicago, IL.

**Keywords:** CSF viral escape, HIV-1, cerebrospinal fluid, drug resistance mutations, antiretroviral therapy

## Abstract

**Background:**

Cerebrospinal fluid (CSF) viral escape is an increasingly recognized clinical event among HIV-1-infected adults. We analyzed longitudinal data and drug-resistance mutations to characterize profiles of HIV-1-infected patients on antiretroviral therapy with discordant CSF and plasma HIV-1 RNA levels.

**Methods:**

Forty-one cases of CSF escape defined as detectable CSF HIV-1 RNA when plasma levels were undetectable, or HIV-1 RNA >0.5-log higher in CSF than plasma were identified from Boston Hospitals and National NeuroAIDS Tissue Consortium (NNTC) from 2005 to 2016.

**Results:**

Estimated prevalence of CSF escape in Boston and NNTC cohorts was 6.0% and 6.8%, respectively; median age was 50, duration of HIV-1 infection 17 years, CD4 count 329 cells/mm^3^ and CD4 nadir 21 cells/mm^3^. Neurological symptoms were present in 30 cases; 4 had repeat episodes of CSF escape. Cases were classified into subtypes based plasma HIV-1 RNA levels in the preceding 24 months: high-level viremia (1000 copies/mL), low-level viremia (LLV: 51–999 copies/mL), and plasma suppression with CSF blip or escape (CSF RNA <200 or ≥200 copies/mL). High-level viremia cases reported more substance abuse, whereas LLV or plasma suppression cases were more neurosymptomatic (81% vs. 53%); 75% of repeat CSF escape cases were classified LLV. M184V/I mutations were identified in 74% of CSF samples when plasma levels were ≤50 copies per milliliter.

**Conclusions:**

Characteristics frequently observed in CSF escape include HIV-1 infection >15 years, previous LLV, and M184V/I mutations in CSF. Classification based on preceding plasma HIV RNA levels provides a useful conceptual framework to identify causal factors and test therapeutics.

## Abstract 2

Supplemental Digital Content is Available in the Text.

## INTRODUCTION

The global prevalence of HIV-1 associated dementia has declined with suppressive antiretroviral therapy (ART), yet milder forms of HIV-1 associated neurocognitive disorders persist in 25%–50% in people with chronic HIV-1 infection.^1^ Factors influencing cognitive outcomes in HIV-1 infected adults on ART include persistent immune activation, genetic predisposition, and irreversible central nervous system (CNS) injury before initiation of ART.^1,2^ HIV-1 infects the brain early after acute infection, and effective ART reduces plasma and cerebrospinal fluid (CSF) HIV-1 RNA levels to below detection limits. Although CSF viral suppression is maintained in most HIV-infected individuals taking ART, higher HIV-1 RNA levels in CSF than plasma are observed in some ART-experienced individuals, a discordance referred to as CSF viral escape.^3,4^

The prevalence of CSF viral escape is estimated at 4%–21% among ART-experienced HIV+ adults.^5–9^ Predictors of CSF escape remain poorly defined; potential risk factors include poor CNS penetration by some ART regimens,^7^ persistent low-level viremia (LLV),^8,9^ length of time on ART,^5,6^ drug-resistance mutations (DRMs) in CSF,^6,8,10,11^ and low CD4 nadir.^3,6,8^ The relationship between CSF escape and neurological symptoms is unclear among individuals on suppressive ART regimens because CSF HIV-1 RNA levels do not correlate consistently with neurological symptoms.^5,8,12^ A designation of asymptomatic CSF viral escape has been proposed to classify individuals with CSF HIV-1 RNA up to 200 copies per milliliter, and absence of new or progressive neurological impairment.^4,5,13^ In contrast, several case series ^3,6–8^ and case reports ^14–29^ describe individuals presenting with new neurological symptoms, discordant elevation in CSF HIV-1 RNA, and substantial improvement after switching ART regimens; many cases have unique DRMs in the CSF. However, the mechanisms leading to CSF viral escape are not well defined.

Given variable prevalence and few published cohorts of CSF viral escape, the aim of this study was to analyze temporal patterns and relationships between CSF viral escape and antecedent plasma HIV-1 RNA levels in a cohort of ART-experienced HIV-1-infected individuals. We also evaluated drug resistance mutations in the viral reverse transcriptase (RT), protease, and integrase genes in plasma and CSF from CSF escape cases from the study cohort and 19 published studies.

## METHODS

### Data Source

This is a retrospective case-series of 41 cases from 2 sources: (1) neurology and infectious disease clinics from 5 Boston hospitals [Massachusetts General Hospital (MGH), Brigham and Women's Hospital (BWH), Beth Israel Deaconess Medical Center, Boston Medical Center and Tufts Medical Center (Tufts MC)]; and (2) NNTC (sites at University of Texas-Galveston, University of California-San Diego, University of California-Los Angeles, and Mount Sinai Medical Center-New York). The NNTC protocol includes neuromedical and cognitive examinations, assessments of comorbidities, and laboratory values for medical, immunologic, and virologic parameters.^30^ NNTC evaluations yield neuropsychological diagnosis of asymptomatic neurocognitive impairment , mild neurocognitive disorder , HIV-associated dementia (HAD), and neuropsychological impairment due to other factors based on Frascati guidelines.^31^ The study was reviewed and approved by the institutional review boards at MGH, BWH, Dana Farber Cancer Institute, and Tufts MC. The institutional review board at each of the 4 NNTC clinical sites approved the research, and subjects signed a written statement of informed consent.

### Study Population

The study was restricted to the calendar period between January 2005 and May 2016. Cases were eligible for inclusion if they were on stable ART regimens for at least 6 months and met either of the following criteria: (1) CSF HIV-1 RNA >50 copies per milliliter when paired plasma HIV-1 RNA were <50 copies per milliliter; or (2) CSF HIV-1 RNA >0.5 log higher than paired plasma levels. NNTC cases were identified by searching the database for subjects with paired plasma and CSF HIV-1 RNA values (within 7 days) (n = 426) before applying the above criteria for CSF escape (n = 29). Boston cases were identified by directly querying HIV providers. A neurological examination was performed in all cases; neurologically symptomatic conditions were defined as symptoms or diagnoses attributable to the CNS, as described.^4^

### Laboratory Measurements and CNS Penetration Effectiveness Calculation

HIV-1 RNA levels were measured in cell-free CSF and plasma at local sites; because of variability by hospital and calendar year, levels reported as undetectable were imputed to the lower limit of detection for the assay (Supplemental Digital Content, Table 1, http://links.lww.com/QAI/A991). The 2010 CNS penetration effectiveness (CPE) score for drug regimens was calculated for each ART regimen.^32^

### HIV Genotypes in Published Studies

Published studies between January 2005 and June 2016 were included if paired plasma and CSF viral load were available, CSF viral load met study criteria for viral escape, and HIV-1 genotype was reported.

### Statistics

Descriptive statistics [means, medians,SD, interquartile range (IQR), percentages, and frequencies] were performed based on variable distribution. Univariate/bivariate tests were conducted using *t* tests, Wilcoxon rank sum tests, or Fisher exact tests in R (version 3.3.0).

## RESULTS

Among Boston and NNTC patients, 41 HIV-1-infected adults met inclusion criteria for CSF viral escape and were included in these analyses. Twenty-nine CSF escape cases were identified from 426 cases with paired plasma and CSF HIV-1 RNA values from the NNTC, a prevalence of 6.8%. Twelve CSF escape cases were identified from the Boston hospitals from approximately 200 patients screened for CSF HIV-1 RNA levels during the study period, an estimated prevalence of 6.0%.

Clinical characteristics for the NNTC and Boston cases are shown in Table 1. The mean age at presentation for the combined cohort was 50 years (SD: 9); 33 men and 8 women. Forty-six percent were non-Hispanic white, and 32% were black. There were no significant differences in age (*P* = 0.4), sex (*P* = 0.2), or race (*P* = 0.5) between the 2 cohorts. The NNTC cohort had higher percentages reporting cocaine and/or amphetamine use (43% vs. 8%) and alcohol use (24% vs. 17%) than the Boston cohort. The median duration of HIV-1 infection in the combined cohort was 17 years (IQR: 13–22), median CD4 T-lymphocyte count 329 cells/mm^3^ (IQR: 217–388), and median CD4 nadir 21 cells/mm^3^ (IQR: 2–60). ART regimens at the time of CSF escape consisted of a ritonavir-boosted protease inhibitor (PI/r) in 31 of 41 cases (76%); PI/r regimens had either at least 2 nucleoside reverse transcriptase inhibitors (NRTIs; n = 29) or an integrase inhibitor (Supplemental Digital Content, Table 2, http://links.lww.com/QAI/A991).

**TABLE 1.**
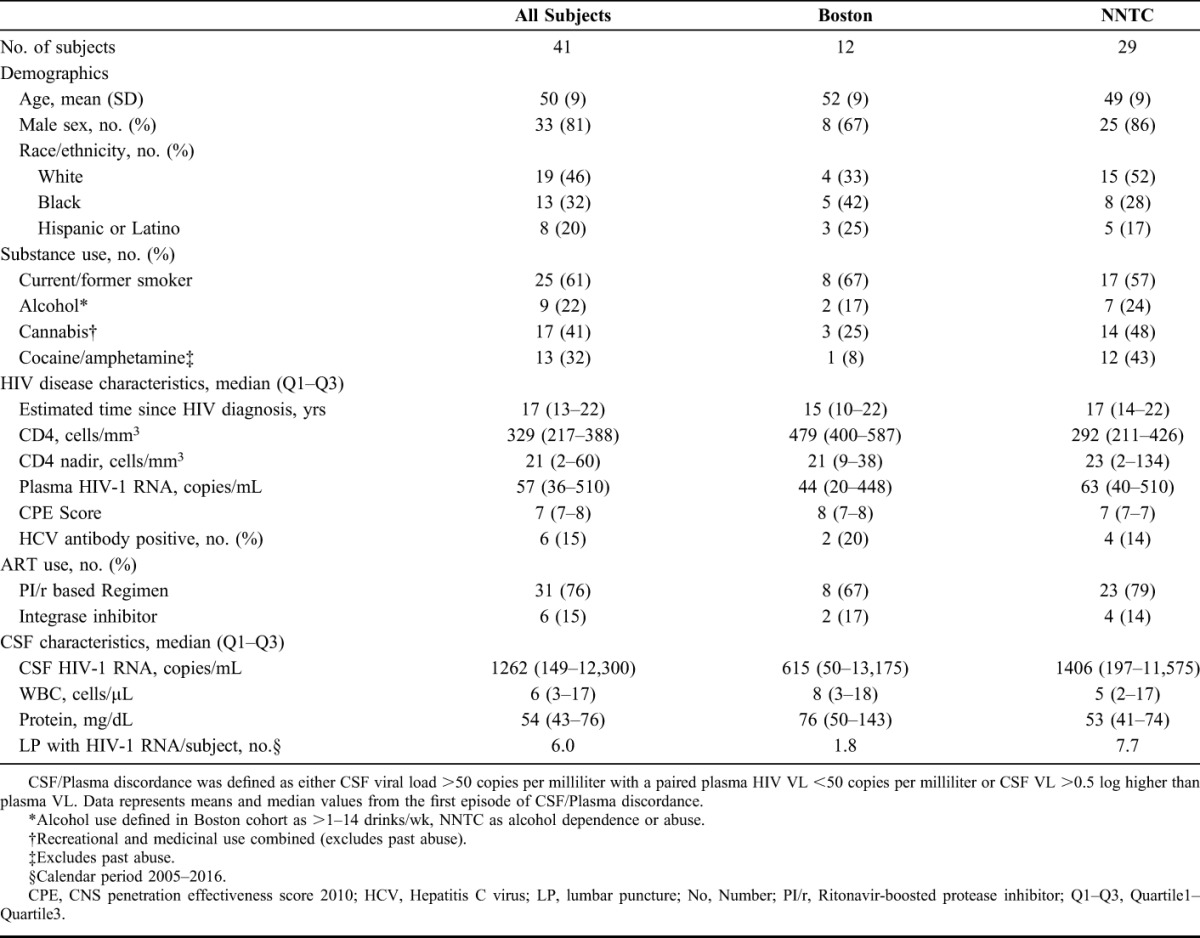
Demographic and HIV-Related Data From the Boston and NNTC Cohorts

Neurological abnormalities included worsening depression, cognitive symptoms, and/or evidence of HIV-1 associated neurocognitive disorders (n = 24), seizures (n = 3), sensory hypoasthesia and carotid artery dissection (n = 1), and stroke (n = 1); 2 cases had a concurrent diagnosis of progressive multifocal leukoencephalopathy and one had CNS lymphoma (Supplemental Digital Content, Table 2, http://links.lww.com/QAI/A991).

All Boston cases had CSF analysis for neurological symptoms, and on average underwent 2 lumbar punctures. The NNTC collects data for clinical and research purposes; 62% of NNTC cases had neurological symptoms at time of lumbar puncture, and on average, each case underwent 7 lumbar punctures. Median CSF HIV-1 RNA levels among NNTC subjects were 1406 vs. 615 copies per milliliter for Boston cases (*P* = 0.4).

**FIGURE 1.**
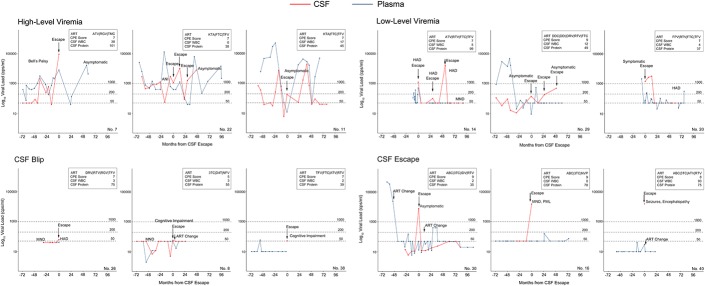
Representative plasma (blue) and CSF (red) HIV-1 RNA levels in patients with persistent high-level or low-level viremia (top panels), CSF blips, or CSF escape (bottom panels). Patients with low-level viremia for at least 6 months, and those with durable plasma suppression for up to 24 months prior to CSF blips (<200 copies/mL) or CSF HIV-1 RNA escape (≥200 copies/mL) were more likely to report neurological symptoms at the time of CSF analysis than patients with high-level viremia. ART, antiretroviral therapy; ANI, asymptomatic neurocognitive impairment; HAD, HIV-associated dementia; MND, mild neurocognitive disorder; PML, progressive multifocal leukoencephalopathy.

Repeat episodes of CSF escape were observed in 4 cases (No. 14, 21, 29, 36; Supplemental Digital Content, Table 2, http://links.lww.com/QAI/A991); 1 case remained neurologically asymptomatic (No. 29). In case 14 (HAD), consecutive CSF HIV-1 RNA was 1262, 101, and 171,000 copies per milliliter at CSF escape, 3 and 4.5 years later, respectively, whereas case 29 (asymptomatic) had CSF HIV-1 RNA of 141, <50, 180, and 490 copies per milliliter, at CSF escape, 1, 2, and 4 years later (Fig. 1). In case 21 (HAD), CSF HIV-1 RNA was 149 copies per milliliter at CSF escape and 1312 copies per milliliter 2 years later, and in case 36 (stroke), CSF HIV-1 RNA was 53 and 45 copies per milliliter 3 years later (not shown); neopterin level in this case was elevated at 45 nmol/L (normal: 8–28 nmol/L) at the second instance of CSF escape.

### Classification of CSF Viral Escape Based on Preceding Plasma HIV-1 RNA Levels

After analyzing all available antecedent plasma HIV-1 RNA levels and their respective relationship to CSF HIV-1 RNA, 4 patterns were evident within 2 broad categories defined by plasma HIV-1 RNA levels (Supplemental Digital Content, Table 3, http://links.lww.com/QAI/A991): incomplete plasma suppression (n = 24) and durable plasma suppression (n = 17). Among cases with incomplete plasma suppression, those with detectable plasma level ≥1000 copies per milliliter or levels between 51 and 999 copies per milliliter during 6 months before escape were defined as high-level viremia (HLV) or LLV, respectively, a cutoff used to differentiate between persistant LLV and virologic failure as previously described.^33–35^ Cases with durable plasma suppression (undetectable plasma HIV-1 RNA levels for 24 monthsbefore escape) were further subdivided as CSF HIV-1 RNA levels <200 copies per milliliter (CSF blip), or levels ≥200 copies per milliliter (CSF escape). Cases with a single viral blip <1000 HIV RNA copies per milliliter 7–24 monthsbefore escape with subsequent return to undetectable levels were considered durably suppressed.^36^ Plasma and CSF HIV-1 RNA levels and timing of neurological symptoms from representative cases for each CSF viral escape subtype (HLV, LLV, CSF blip, and CSF escape) are depicted in Figure 1.

Among 24 cases with incomplete plasma suppression, notable findings included 67% of HLV cases reporting cocaine and/or amphetamine abuse at time of presentation, a higher proportion than for LLV or durable plasma suppression cases (Table 2). The median calendar year at time of presentation for HLV was 2007, historically earlier than cases classified as LLV (2012), CSF blip (2013), or CSF escape (2011). There were no differences between HLV and LLV cases for duration of HIV infection (15 vs. 18 years; *P* = 0.3), plasma HIV-1 RNA copies at CSF escape (400 vs. 258 copies/mL; *P* = 0.1), or CD4 nadir (34 vs. 14 cells/mm^3^; *P* = 0.7); CD4 cell count was lower in the HLV subgroup (*P* = 0.04). Although 6 of 9 LLV subjects had multiple visits with plasma HIV-1 RNA levels between 51 and 999 copies per milliliterbefore CSF escape, in 5 cases, CSF HIV-1 RNA levels were >1 log_10_ higher than the preceding 12 months of plasma HIV-1 RNA levels. Paired plasma and CSF HIV-1 genotypes were available for 3 LLV cases (Table 3). Although most mutations in plasma and CSF were similar, 2 of 3 LLV cases had unique mutations in CSF (case 1: D67N, N348I, E399D, K20R; case 2: P225L, T74A). Three of 4 cases with repeat escape (No. 14, 21, 29) were classified as having LLV 6 months before the first instance of escape.

**TABLE 2.**
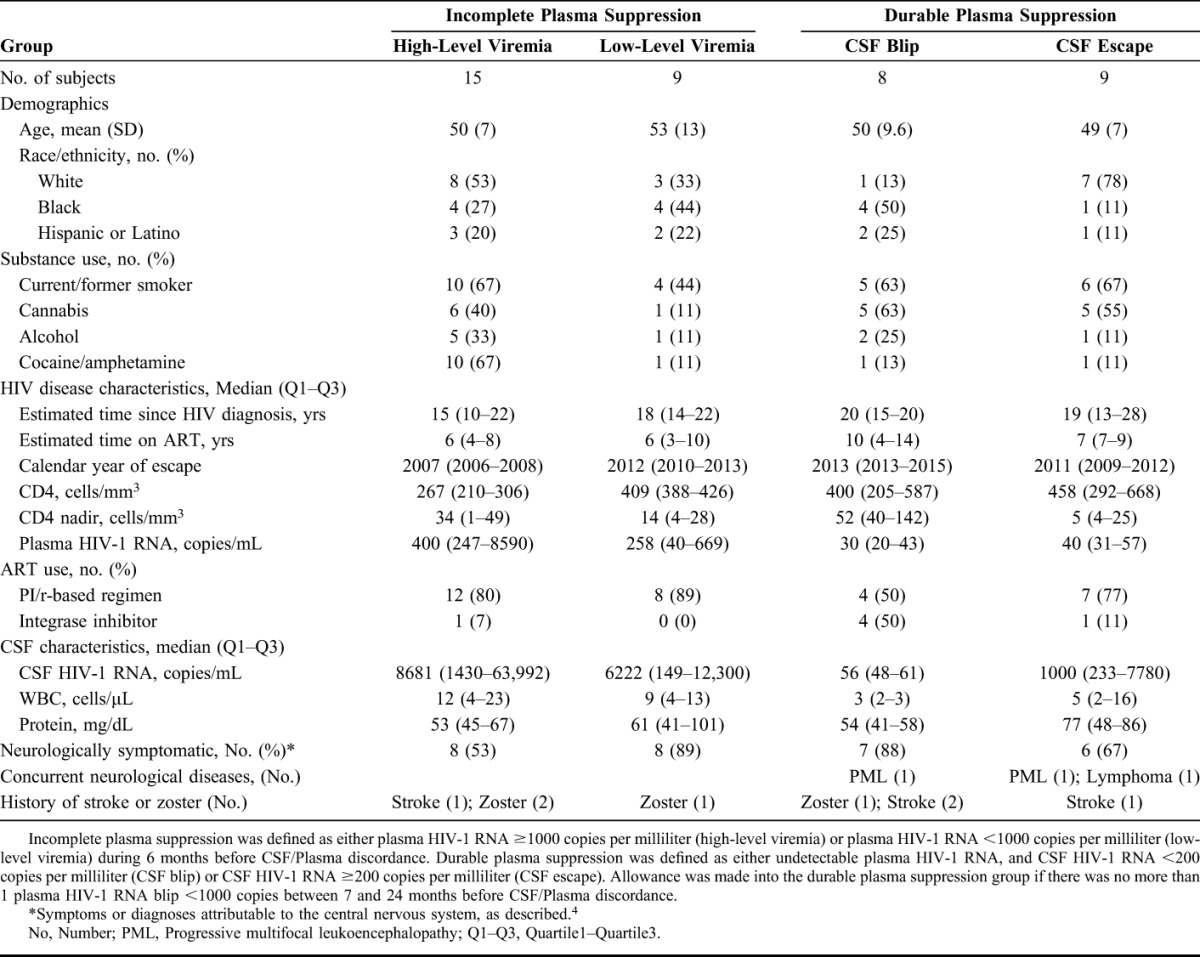
Demographic and HIV-Related Data by CSF/Plasma Discordance Subtypes

**TABLE 3.**
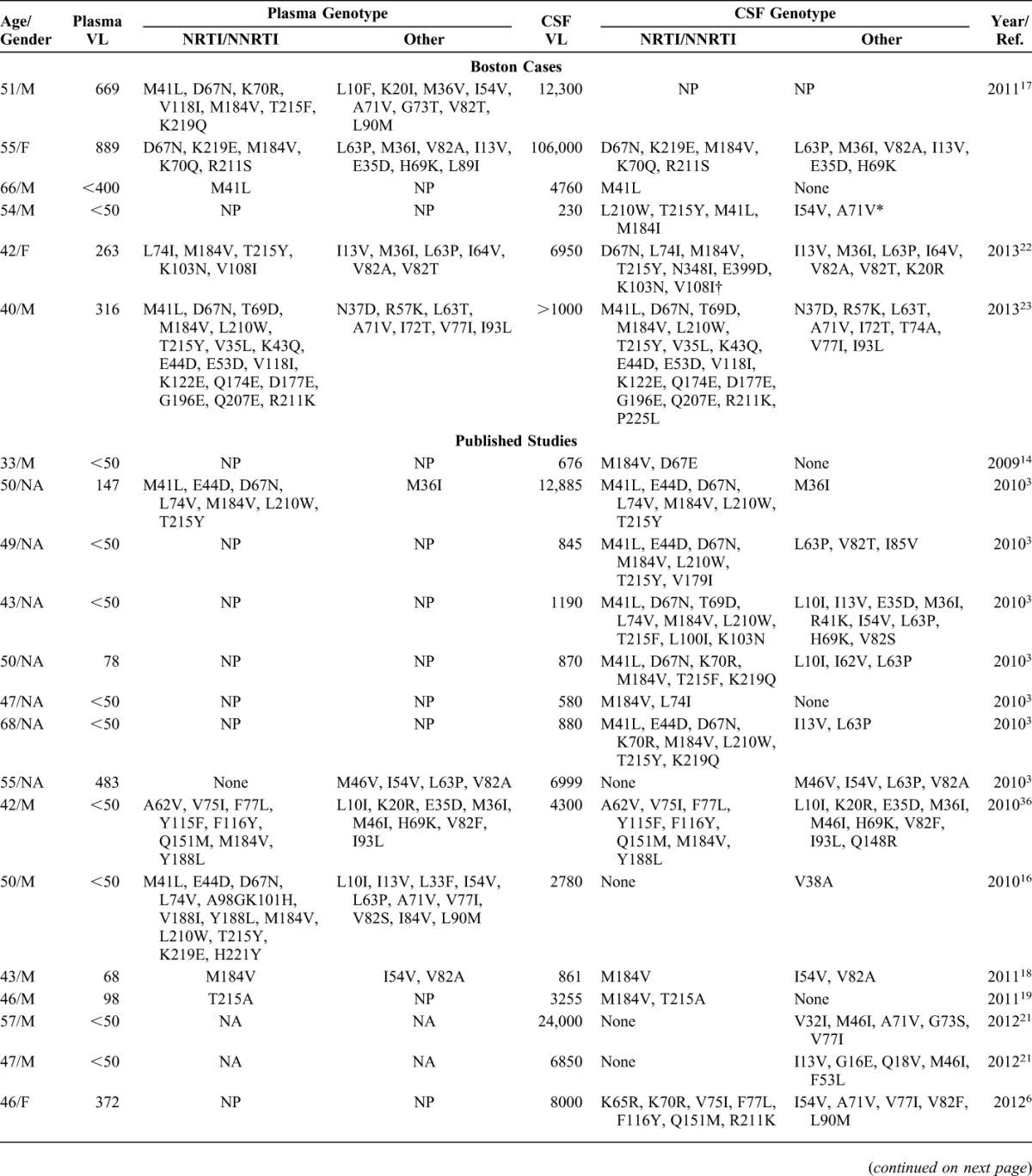

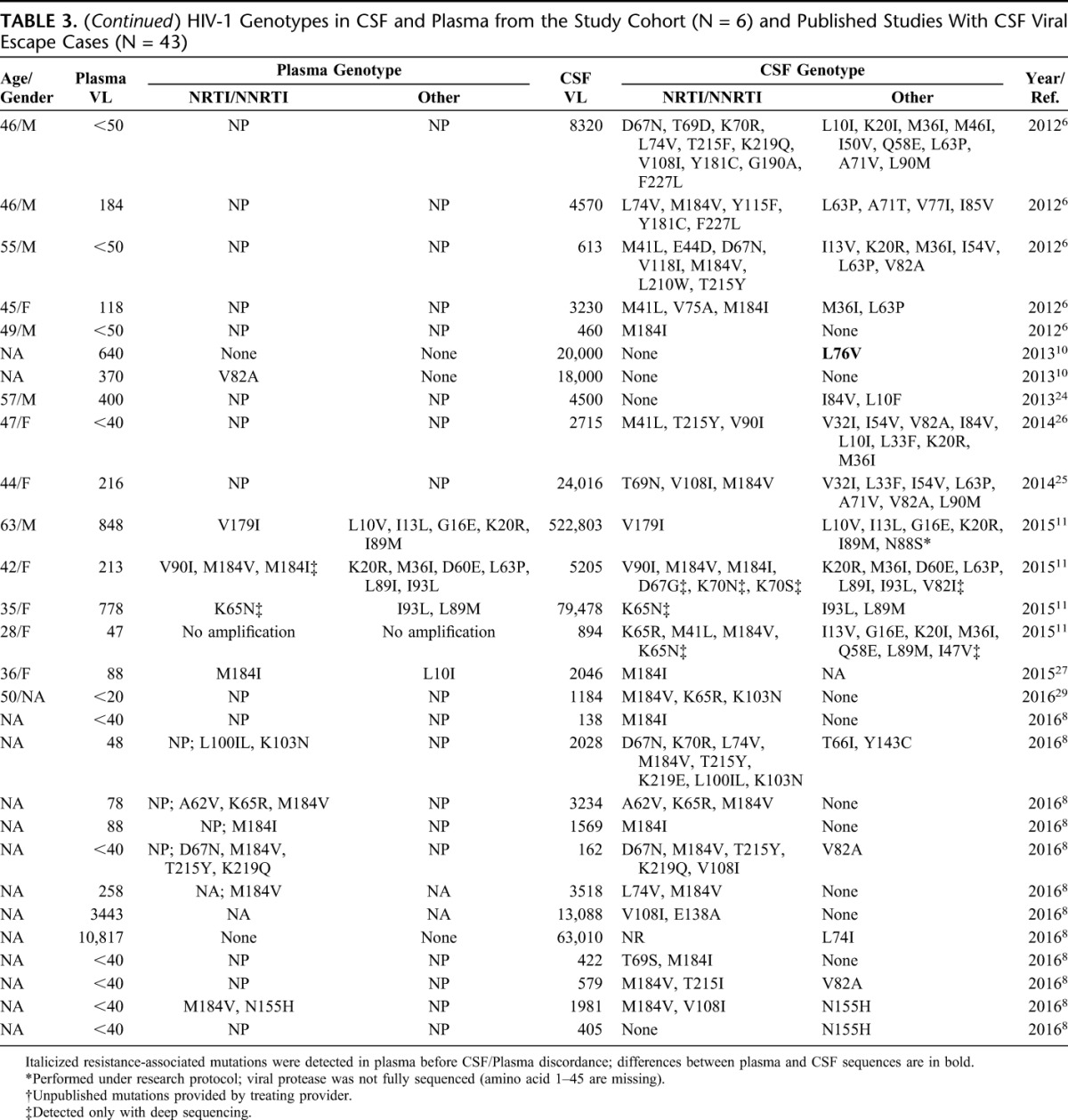
HIV-1 Genotypes in CSF and Plasma from the Study Cohort (N = 6) and Published Studies With CSF Viral Escape Cases (N = 43)

Among 17 cases with durable plasma suppression, subgroups classified as CSF blip vs. CSF escape were similar with respect to age, duration of HIV diagnosis, plasma HIV-1 RNA levels, and CD4 cell count (Table 2). CSF HIV-1 RNA levels were lower in CSF blip versus CSF escape subgroups (56 vs. 1000 copies/mL; *P* = 0.001), whereas CSF WBC (*P* = 0.2) and CSF total protein levels (*P* = 0.1) were similar. One case classified as CSF escape had multiple thymidine analogue-associated mutations (TAMs; L210W, T215Y, and M41L) in the presence of the M184I mutation (Table 3) on a regimen of abacavir, lamuvidine, and ritonavir-boosted lopinavir, effectively resulting in PI/r monotherapy in the CNS compartment (No. 12; Supplemental Digital Content, Table 2, http://links.lww.com/QAI/A991).

### Plasma and CSF HIV-1 Genotypes at the Time of CSF Viral Escape

Given that drug-resistant HIV-1 virus has been frequently associated with CSF viral escape, we analyzed HIV-1 mutations from this cohort and published studies. A total of 172 mutations in the RT gene, 135 mutations in the protease gene and 6 mutations in the integrase gene were identified in CSF from 49 cases (Table 4). Fourteen of 49 cases with CSF escape had paired plasma and CSF HIV-1 genotypes (29%) with D67N/G reported as a unique CSF mutation twice. Most plasma genotypes were not reported because of undetectable plasma HIV-1 RNA or levels too low to be amplified. The most common CSF mutations in RT were M184V and M184I (19% of all CSF mutations identified). M184V/I mutations were present in CSF isolates among 17 of 23 cases (74%) with plasma HIV RNA ≤50 copies per milliliter. TAMs were present in 20 of 49 cases (40%), most commonly T215Y, D67N, or M41L (Table 4). The most common PI mutations were V82A/T/F/S/I; V32I mutation was present in 3 cases, and has been associated with high level of resistance in combination with other PI mutations.^37,38^ Two studies reported sequencing the integrase gene in CSF^8,39^; N155H, L74I, and Q148R mutations were observed as single mutations in the integrase gene, and Y143C was observed in combination with T66I.

**TABLE 4.**
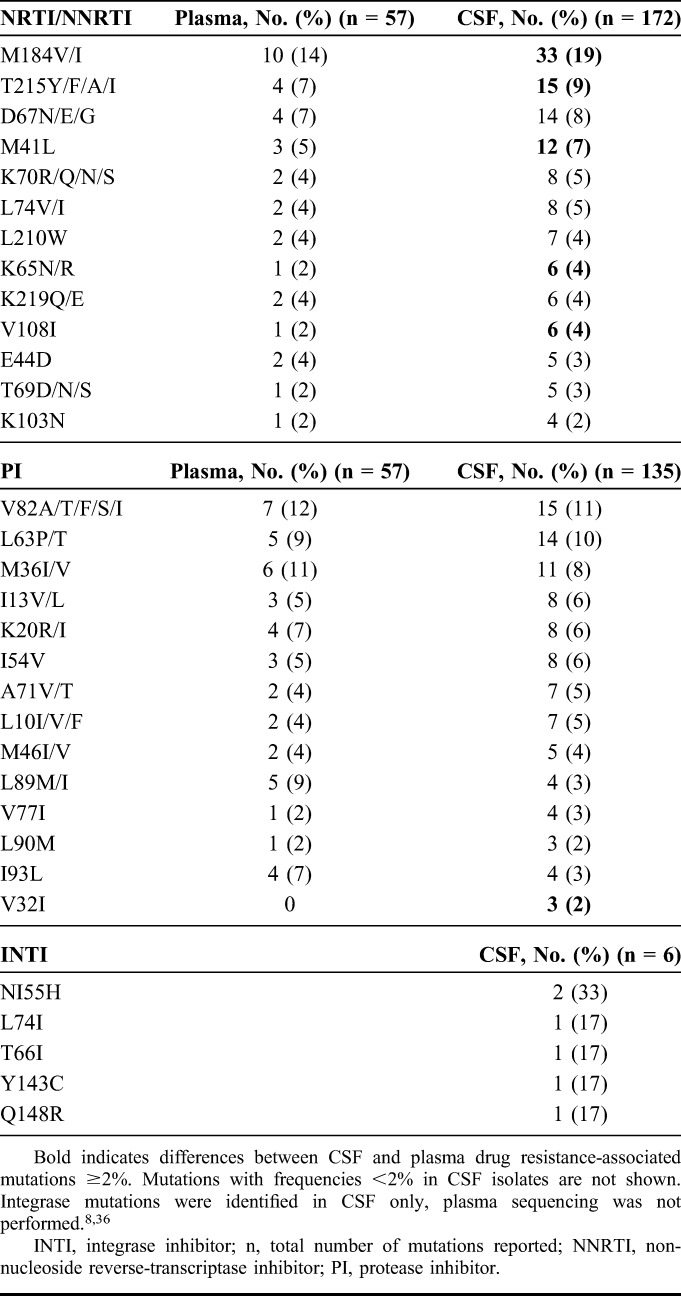
Frequencies of HIV-1 Mutations in CSF Viral Escape Cases

## DISCUSSION

This longitudinal analysis of 41 ART-experienced HIV+ individuals with CSF viral escape is one of the largest case-series investigating an uncommon, but increasingly recognized clinical event among ART-treated HIV+ individuals. The estimated prevalence and clinical characteristics of CSF escape were similar between Boston hospitals (6.0%) and NNTC clinical research cohort (6.8%). Four cases had detectable CSF HIV-1 RNA at multiple time points despite plasma suppression, indicative of repeated CSF escape, a phenotype that is not yet well characterized in the literature.^13^ Cases in this study cohort were classified into 4 subtypes based on plasma HIV RNA levels in the preceding 24 months: HLV(≥1000 copies/mL), LLV(51–999 copies/mL), and plasma suppression with CSF blip or escape (CSF HIV-1 RNA <200 or ≥200 copies/mL). Temporal analysis of antecedent plasma viral loads revealed that cross-sectional definitions of viral escape based on >0.5 log_10_ higher levels of CSF HIV-1 RNA will include HLV cases, a subtype with sustained plasma viremia. In HLV cases, discordant CSF viral levels could reflect delayed CSF viral clearance or continuous CSF seeding from plasma,^40^ and therefore, may not necessarily represent compartmentalized virus. In contrast, definitions requiring plasma suppression will miss LLV cases, a subtype previously shown to harbor DRMs in CSF,^8^ and here shown to have repeat episodes of viral escape. M184V/I were the most frequently observed mutations in all cases present in 74% of CSF isolates from individuals with plasma levels ≤50 copies per milliliter, and frequently occurring in the presence of other DRMs.

By classifying CSF viral escape based on plasma HIV-1 RNA in the 24 months before CSF discordance, we identified differences in clinical characteristics associated with CSF escape subtypes. HLV cases were more likely to use cocaine, amphetamines, and alcohol, behaviors that are frequently linked to suboptimal ART adherence and reduced health care engagement.^41^ HLV cases also made up a greater proportion of viral escape casesbefore 2010, whereas durable plasma suppression and detectable CSF HIV-1 RNA classified cases represented most CSF escape cases after 2010. This shift in calendar period could represent a change in clinical practices (ie, increased CSF HIV-1 RNA testing for neurological symptoms) because of recent guideline recommendations,^42–44^ increased use of ultrasensitive HIV-1 RNA assays and/or decreased prevalence of virologic failure because of improved ART regimens. Although low levels of CSF HIV-1 RNA may reflect variability in assay sensitivity and be unrelated to neurological symptoms, a recent study showed that low CSF HIV-1 RNA levels in viral escape were associated with elevated inflammatory mediators and endothelial adhesion molecule VCAM,^45^ and may not always be benign. One factor that can account for the association with neuroinflammation is secondary escape, defined as discordant CSF and plasma HIV-1 RNA levels in the context of a new infection.^4^ In our series, there were 3 candidates for secondary escape; 2 cases with progressive multifocal leukoencephalopathy and one with CNS lymphoma. As the field begins to harmonize clinical definitions for CSF escape, the epidemiology and associated cofactors of escape subtypes based on previous plasma HIV-1 RNA levels will be a relevant indicator of evolving clinical practices, and may also enable future studies to identify biomarkers linked to virologic failure in the CNS.

HIV-1 has high genetic diversity and recombination, which can result in DRMs when viral replication is incompletely suppressed because of factors such as poor adherence, persistent LLV, and incomplete ART penetration into the CNS. In the present study, over 80% of individuals had HIV-1 infection for greater than 10 years before CSF escape with long exposures to ART, including older regimens, suggesting duration of HIV-infection, ART use, and potential emergence of DRMs are likely contributing factors to CSF escape. Although there has been a declining trend in resistance mutations among HIV+ individuals in resource-rich settings,^46^ a 2015 study in ART-experienced patients with plasma HIV-1RNA >50 copies per milliliter showed that 12.5% and 19.8% of individuals had unique CSF resistance mutations to RT and protease, respectively; M41L and T215Y, were statistically more prevalent in the CSF.^47^ In our study, M184V/I, T215Y, D67N, and M41L were the most commonly detected CSF mutations. Three CSF integrase mutations were identified in CSF (N155H, Y143C, and Q148R). Although these mutations are not known to reduce susceptibility to integrase inhibitors in isolation,^38^ integrase mutations remain important to monitor.

A recent study showed that low-level of CSF HIV-1 RNA was associated with better neurocognitive outcomes, and hypothesized that one mechanism was through DRMs such as M184V, which reduce viral fitness but may stimulate a more effective immune response.^9^ However, the combination of poor CSF penetration by tenofovir, a widely used NRTI in ART^48^ and M184V/I mutation leading to emtricitabine or lamuvidine resistance may lower the threshold for subsequent CSF mutations that could lead to NRTI cross resistance.^38^ The high frequency of M184V/I mutations reported here and in the literature, and simultaneous presence of TAMs in neurologically symptomatic CSF escape raises the possibility that the M184V/I mutations could be a factor influencing CNS viral reservoirs and CSF escape. Future studies that systematically assay CSF DRMs, particularly in neurosymptomatic individuals, will help to understand the impact of M184 and other DRMs on CNS viral reservoir size, their potential impact on neuropathogenesis, and effects on neurological outcomes.

Persistent LLV is associated with plasma virologic failure, and resistance mutations arising from stable HIV-1 reservoirs or ongoing replication cycles may influence viral pathogenesis in the CNS.^8,45,49^ Three of 4 cases in this series with historical evidence of LLV in plasma had recurrent CSF escape; most but not all were associated with neurological symptoms. In a recent series of longitudinal CSF samples from 75 neurologically asymptomatic HIV+ individuals on ART,^13^ 7 cases had a quantifiable CSF HIV-1 RNA in 2 consecutive samples and remained neurologically asymptomatic.^13^ These data suggest that dynamic CSF viremia is present among symptomatic and asymptomatic individuals, and research efforts to identify individuals with CNS viral reservoirs may require CSF profiling irrespective of neurological symptoms.^8^ An additional consideration is CNS penetration of ART regimens as calculated by CPE score. An “adjusted” CPE score has been proposed as a more relevant score in CSF escape, as it takes into account resistance profiles for the calculation of CNS ART effectiveness.^6^ Although CPE score at the time of escape was not a distinguishing feature in this series, 6 cases with genotyping from CSF or plasma isolates had mutations that would result in resistance to at least one prescribed ART drug, resulting in a lower “adjusted” CPE score. Together, these findings support the strategy that changes in treatment regimens for CSF escape should be guided by CSF resistance testing, when possible.^42–44^

This study has several limitations common to retrospective case-series including selection and recall bias, and limitations in data based on chart reviews. In addition, there was no systematic cataloging of CNS escape in the Boston cohort; thus, identification of these cases relied on current providers' recollection of patients, which has the potential to skew analysis toward more recent years and cases with severe neurological symptoms and may lead to underreporting true prevalence. Given that HIV-1 RNA assays are not FDA-approved for CSF quantification and testing has only recently been adopted in US-based guidelines, ^42,43^ there is considerable heterogeneity in provider practices. As such, neurological symptoms requiring hospitalization or profound cognitive decline are more likely to lead to CSF HIV-1 RNA quantification, whereas milder neurological symptoms may not result in lumbar punctures unless imaging abnormalities are identified.

The precise definitions of CSF viral escape, neurological symptoms that require CSF analysis, and when providers should repeat lumbar puncture after CSF viral escape remains debated. Our estimates suggest that individuals with a recent history of LLV or historical virologic failure with M184V/I mutations are potentially at elevated risk for CSF escape. Given that the search for a curative strategy is a top priority, barriers to cure including LLV on ART and replication-competent HIV-1 virus in the CNS require further study.^50^ The CSF escape subtypes we propose will help to shed light on the true prevalence, and provide a new conceptual framework for future studies of viral and host factors leading to CSF escape and testing of therapeutics aimed at virus eradication.

## Supplementary Material

SUPPLEMENTARY MATERIAL
