# Supplementary material for: Temporal Patterns and Drug Resistance in CSF Viral Escape Among ART-Experienced HIV-1 Infected Adults
Source: J Acquir Immune Defic Syndr. 2017 May 16;75(2):246–55. doi: 10.1097/QAI.0000000000001362 (PMC5452976; doi:10.1097/QAI.0000000000001362)
Supplement: SUPPLEMENTARY MATERIAL [file qai-75-246-s001.docx]

**Supplementary Table 1**: HIV-1 RNA Assays Used

| **Cohort** | **HIV-1 RNA Assay** | **Limit of Detection (copies/ml)** |
| --- | --- | --- |
| NNTC | Roche HIV-1 Amplicor Monitor v.1.0/v.1.5 ultrasensitive | <20 |
|  | Roche HIV-1 Amplicor Monitor v.1.0/v.1.5 standard sensitivity | <400 |
|  | Abbott m2000 Real-Time PCR System | <40 |
|  | bDNA (Chiron, Bayer) | <75 |
| Boston Hospitals | Roche TaqMan HIV-1 Test v1.0/v2.0 | <20 |
|  | Abbott m2000 Real-Time PCR System | <40 |

**Supplementary Table 2:** Detailed Clinical Characteristics of Subjects with CSF/Plasma Discordance

| No. | Age/Gender | Plasma VL | CSF VL | CSF WBC | ART | Neurological Signs/Symptoms/Diagnosis^α^ | Regimen Change | Post-Change Symptoms |
| --- | --- | --- | --- | --- | --- | --- | --- | --- |
| **1** | 40/M | 75 | 1000 | 175 | ATV\|RTV\|FTC\|TFV | Dizziness, Seizures, Vertigo, Diplopia | MVC\|DRV\|RTV\|RGV | Improved Cognition |
| **2** | 53/M | <40 | 233 | 4 | 3TC\|RGV\|ABC | CNS Lymphoma |  |  |
| **3** | 45/M | 400 | 8681 | 12 | ATV\|DDI\|RTV\|TFV | None |  |  |
| **4** | 61/M | <40 | 295 | 1 | DRV\|RTV\|FTC\|TFV | None |  |  |
| **5** | 59/M | 9226 | 40676 | 17 | DRV\|RTV\|FTC\|TFV | None |  |  |
| **6** | 50/F | <20 | 37 | 3 | ABC\|3TC\|DTG | Sensory hypoesthesia/Carotid Artery Dissection |  |  |
| **7** | 45/M | 8301 | 94395 | 38 | ATV\|RGV\|ETR | None |  |  |
| **8** | 40/M | <50 | 59 | 3 | 3TC\|D4T\|NFV | NPI-O | FTC\|NFV\|TFV | MND |
| **9** | 51/M | 1118 | 11575 | 17 | FPV\|RTV\|FTC\|TFV | ANI |  |  |
| **10** | 67/M | 36 | 123 | 10 | EFV\|FTC\|TFV | ANI |  |  |
| **11** | 40/M | 12 | 179 | 2 | LPV\|RTV\|FTC\|TFV | None |  |  |
| **12** | 54/M | <50 | 230 | 16 | 3TC\|ABC\|LPV\|RTV | Cognitive Changes, Fatigue, Depression, Tremor | FTC\|TDF\|DRV\|RTV\|ETR\|RGV | Improved Cognition and Fatigue |
| **13** | 71/M | 258 | 6222 | 6 | DRV\|RTV\|FTC\|TFV | NPI-O |  |  |
| **14** | 43/M | 510 | 1262 | 5 | ATV\|RTV\|FTC\|TFV | HAD |  |  |
| **15** | 40/M | 255 | 4254 | 23 | ATV\|RTV\|FTC\|TFV | ANI |  |  |
| **16** | 48/M | <50 | 15052 | 0 | ABC\|3TC\|NVP | MND (PML) |  |  |
| **17** | 46/M | 48335 | 573054 | 40 | 3TC\|D4T\|LPV\|RTV | MND |  |  |
| **18** | 66/M | <20 | 72 | 10 | ABC\|3TC\|ATV\|RTV | Cognitive Changes | ABC\|3TC\|DRV\|RTV | Improved Executive Function |
| **19** | 42/F | 7086 | 44288 | 4 | ATV\|RTV\|FTC\|TFV | HAD |  |  |
| **20** | 36/F | <50 | 1406 | 3 | FPV\|RTV\|FTC\|TFV | None |  |  |
| **21** | 51/F | <40 | 149 | 0 | DRV\|RTV\|FTC\|TFV | HAD |  |  |
| **22** | 40/M | 400 | 1138 | 9 | LPV\|RTV\|FTC\|TFV | None |  |  |
| **23** | 52/M | 239 | 6555 | 32 | 3TC\|RTV\|SQV\|TFV | None |  |  |
| **24** | 41/M | 825 | 17040 | 38 | ATV\|RTV\|FTC\|TFV | ANI |  |  |
| **25** | 51/M | 669 | 12300 | 1 | FTC\|TFV\|LPV\|RTV\|T20 | Cognitive Changes | DRV\|TMC\|RGV\|LPV\|RTV\|T20 | Improved Cognition |
| **26** | 63/M | 41 | 64 | 10 | DRV\|RTV\|RGV\|TFV | HAD |  |  |
| **27** | 53/M | <20 | 28 | 3 | ABC\|3TC\|RGV | Headache, Cognitive Changes |  |  |
| **28** | 47/M | <50 | 294 | 7 | ATV\|ABC\|3TC\|RTV | MND |  |  |
| **29** | 49/M | 9 | 141 | 12 | DDC\|DDI\|DRV\|RTV\|FTC | None |  |  |
| **30** | 55/F | 13 | 7780 | 2 | 3TC\|ABC\|IDV\|RTV | None |  |  |
| **31** | 68/M | 63 | 197 | 4 | FPV\|RGV\|RTV | NPI-O |  |  |
| **32** | 45/M | <40 | 60 | 1 | 3TC\|DRV\|RTV\|ABC | NPI-O (PML) |  |  |
| **33** | 42/M | 264 | 1721 | 3 | EFV\|ZDV\|3TC\|ABC | NPI-O |  |  |
| **34** | 43/M | 27 | 299 | 1 | ATV\|DRV\|RTV\|FTC\|TFV | NPI-O |  |  |
| **35** | 49/M | 23019 | 83696 | 23 | RTV\|SQV\|FTC\|TFV | None |  |  |
| **36** | 49/F | <20 | 53 | 2 | TFV\|FTC\|EFV | Stroke |  |  |
| **37** | 37/F | 263 | 6950 | 13 | ABC\|3TC\|TFV\|ATV\|RTV | Cognitive Changes | RTV\|DRV\|RGV\|ZDV\|3TC\|TFV | Improved Cognition |
| **38** | 61/M | <20 | 52 | 2 | TFV\|FTC\|ATV\|RTV | Cognitive Changes, Depression |  |  |
| **39** | 66/M | 8880 | 750000 | 4 | EFV\|FTC\|TFV | Depression, Seizure, Ataxia | ATV\|RTV\|3TC\|ZDV | Improved attention |
| **40** | 41/M | 31 | 15800 | 93 | ABC\|3TC\|ATV\|RTV | Seizure | ABC\|3TC\|RGV | Improved Speech, Increased Attention |
| **41** | 55/F | 889 | 106000 | 178 | TFV\|FTC\|LPV\|RTV | Cognitive Changes | EVG\|COBI\|TFV\|FTC\|DRV |  |

α Neurological symptoms suggestive of CNS escape as published [^4^](#_ENREF_4); 3TC, Lamivudine; ABC, Abacavir; ANI, asymptomatic neurocognitive impairment; ART, anti-retroviral therapy; ATV, Atazanavir; CSF, cerebrospinal fluid; CNS, central nervous system; D4T, Stavudine; DDC, Zalcitabine; COBI, Cobicistat; DDI, Didanosine; DRV, Darunavir; DTG, Dolutegravir; EVG, Elvitegravir; EFV, Efavirenz; ETR, Etravirine; FPV, Fosamprenavir; FTC, Emtricitabine; HAD, HIV-associated dementia; IDV, Indinavir; LPV, Lopinavir; MND, mild neurocognitive disorder; MVC, Maraviroc; NFV, Nelfinavir; NVP, Nevirapine; NPI-O, neuropsychological impairment, other reasons; PML, progressive mulitfocal leukoencephalopathy; RGV, Raltegravir; RTV, Ritonavir; SQV, Saquinavir; T20, Enfuvirtide; TFV, Tenofovir disoproxil fumarate; WBC, white blood cells; VL, viral load; ZDV, Zidovudine

**Supplementary Table 3:** Definition of CSF HIV-1 Escape Subtypes

| **Incomplete Plasma Suppression** |  |
| --- | --- |
| High-Level Viremia (HLV) | *Detectable plasma VL≥1000 copies/ml during 6 months prior to escape* |
| Low-Level Viremia (LLV) | *Detectable plasma VL <1000 cps/ml during 6 months prior (allow <2500 copies/ml 7-24 months prior)* |
| **Durable Plasma Suppression** |  |
| CSF Blip | *Undetectable plasma VL for 24 months prior to escape (allow 1 plasma blip <1000 7-24 months prior), CSF VL <200 copies/ml* |
| CSF Escape | *Undetectable plasma VL for 24 months prior to escape (allow 1 plasma blip <1000 7-24 months prior), CSF VL ≥ 200 copies/ml* |
